# The effects of fentanyl, oxycodone, and butorphanol on gastrointestinal function in patients undergoing laparoscopic hysterectomy: a prospective, double-blind, randomized controlled trial

**DOI:** 10.1186/s12871-022-01594-9

**Published:** 2022-02-24

**Authors:** Minna Guo, Shijiang Liu, Jian Gao, Chuanbao Han, Chun Yang, Cunming Liu

**Affiliations:** 1 Faculty of Anesthesiology, Changhai Hospital, Naval Medical University, Shanghai, China; 2 Department of Anesthesiology and Perioperative Medicine, The First Affiliated Hospital of Nanjing Medical University, Nanjing, China; 3 School of Public Health, Shanxi Medical University, Taiyuan, China

**Keywords:** Postoperative gastrointestinal tract dysfunction, Opioid receptor, Analgesia

## Abstract

**Background:**

Perioperative opioid use is associated with postoperative bowel dysfunction, which causes longer hospital stay and higher healthcare costs. This study aimed to investigate the effect of the equivalent doses of fentanyl, oxycodone, and butorphanol on bowel function in patients undergoing laparoscopic hysterectomy.

**Methods:**

In this randomized controlled trial, 135 patients undergoing laparoscopic hysterectomy received postoperative intravenous patient-controlled analgesia (IV-PCA) with fentanyl 8.3 μg/kg, butorphanol 0.16 mg/kg, and oxycodone 0.5 mg/kg (1: 20: 60), respectively. The primary outcome measure was the recovery of bowel function. We also evaluated and recorded the following nine indicators: pain score, sedation level, leukocyte count, percentage of neutrophils, plasma potassium levels, time to first ambulation, postoperative side effects, patients' satisfaction, and postoperative hospital length of stay.

**Results:**

The mean time to flatus was significantly prolonged in Group B (45.2 ± 11.6 h) compared with Group F (33.1 ± 11.2 h, *P* < 0.001) and Group O (36.2 ± 10.9 h, *P* = 0.001). The incidence of somnolence and dizziness prove higher in Group B (*P* < 0.001). No statistical difference was observed in the mean time to tolerate oral diet, time to defecation, analgesic outcome, satisfaction score, time to first ambulation, and postoperative hospital length of stay.

**Conclusions:**

Compared with fentanyl and oxycodone, butorphanol prolonged the recovery of bowel function with more severe somnolence and dizziness, suggesting that butorphanol is not well suitable for IV-PCA in patients undergoing laparoscopic hysterectomy.

**Trial registration:**

ClinicalTrials.gov-NCT04295109. Date of registration: March, 2020.

## Background

Postoperative gastrointestinal tract dysfunction (PGID) is a frequent occurrence after abdominal surgery, which contributes to patient discomfort and increased healthcare costs [1]. Although the minimally invasive techniques bring less surgical trauma compared to open surgery, most of the patients experience post-laparoscopic pain and call for opioid drugs [2, 3]. However, consumption of opioid drugs may lead to PGID by activating opioid receptors, of which classical opioid receptors constitute mu-opioid receptors (MOR), delta-opioid receptors (DOR), and kappa-opioid receptors (KOR) [4, 5].

Most of the previous studies support that PGID is primarily mediated by MOR [4–6]. Therefore, some investigators tried MOR agonist–antagonist instead of pure MOR agonists to reduce the occurrence of PGID. Butorphanol, a mixed opioid receptor agonist–antagonist, has been shown to better lower the incidence of constipation than morphine, the pure MOR agonist [7]. Other investigators who compared pure MOR agonists with oxycodone, a semisynthetic opioid analgesic, which activates MOR and KOR, demonstrated that oxycodone showed a lower incidence of adverse complications than sufentanil [8, 9]. However, these studies focused on two types of opioid receptor agonists and emphasized the differences between analgesic effects, taking nausea/vomiting, not bowel function, as postoperative gastrointestinal function evaluation criteria [7–9]. The effect of pure MOR agonists, multiple opioid receptor agonists, and MOR agonist–antagonist on PGID remains to be elucidated.

This study aimed to compare the effect of the equivalent doses of fentanyl (pure MOR agonists), oxycodone (multiple opioid receptor agonists), and butorphanol (MOR agonist–antagonist) on the recovery of bowel function in patients undergoing the laparoscopic hysterectomy, thereby providing a clinical reference for fundamental research.

## Methods

This prospective, randomized and double-blind study was approved by the Institutional Review Board of the First Affiliated Hospital of Nanjing Medical University (IRB: 2019-SR-476), and conducted in compliance with local regulatory requirements, Good Clinical Practice (GCP), and the Declaration of Helsinki [10], and written informed consent was obtained from all subjects participating in the trial. The trial was registered prior to patient enrollment at clinicaltrials.gov (NCT04295109, Principal investigator: Cunming Liu, Date of registration: March 4, 2020). This manuscript adheres to the applicable Consolidated Standards of Reporting Trials (CONSORT) guidelines.

### Patients

We enrolled patients aged 40–65 years with the American Society of Anesthesiologists (ASA) physical status I-II undergoing laparoscopic hysterectomy. The exclusion criteria were as follows: history of gastrointestinal surgery or gastrointestinal disease (peptic ulcer disease, Crohn’s disease, or ulcerative colitis, etc.); history of alcohol or opioid abuse; chronic use of opioids; allergy and contraindication to fentanyl, oxycodone or butorphanol, or any of their excipients; diabetes mellitus; severe cardiac/pulmonary/hepatic/renal dysfunction; psychiatric disease; pregnant or breastfeeding women; participants in other drug trials in the past three months. Also, participants were withdrawn for postoperative infection, bleeding and mechanical faults with intravenous patient-controlled analgesia (IV-PCA) device.

### Anesthesia protocols

Each patient was monitored using electrocardiography (EKG), pulse oximetry, capnography, temperature and Bispectral Index (BIS). Also, oxygen saturation (SpO_2_), heart rate (HR) and mean blood pressure (MBP) were recorded every 5 min.

After obtaining HR and MBP, anesthesia was induced with midazolam 0.05 mg/kg, fentanyl 3 µg/kg, etomidate 0.3 mg/kg, dexamethasone 5-10 mg, and phencyclidine hydrochloride 0.5-1 mg, and cisatracurium 0.15 mg/kg as an adjunct to tracheal intubation. Fentanyl 6 μg/kg and flurbiprofen axetil 50 mg were injected intravenously 10 min before skin incision. Propofol (6–8 mg·kg^−1^·h^−1^) and remifentanil (0.05–0.2 μg·kg^−1^·min^−1^) were given intraoperatively to titrate analgesia and keep HR and MBP within 20% of baseline values. Besides, cisatracurium (0.1–0.2 mg·kg^−1^·h^−1^) was used applied to maintain neuromuscular blockaded during surgery. End-tidal carbon dioxide concentration was controlled at 35–45 mmHg (1 mmHg = 0.133 kPa) under 10–12 mmHg pneumoperitoneum. BIS was maintained 40–60. If bradycardia (HR < 45 beats/min) and continuous hypotension (MBP < 20% of the baseline values) persisted, additional fluid infusion, atropine, or ephedrine were administered. Patients were given intravenous granisetron 3 mg near the completion of the procedure.

### PACU protocols

After surgery, all patients were transferred to the post-anesthesia care unit (PACU). Extubation was performed when the extubation criteria were met, and then the IV-PCA device was used.

We used fentanyl, oxycodone or butorphanol in the IV-PCA device. Among them, we chose fentanyl group as the active-controlled group. Because fentanyl is one of the most widely used opioids for intravenous patient-controlled analgesia (IV-PCA) and has fewer side effects [11–14]. Previous studies have shown a fentanyl-to-oxycodone conversion ratio of 1:55–100 [15, 16]. However, when the fentanyl-oxycodone ratio is ≥ 1:80, most patients cannot tolerate the high incidence of nausea and vomit so analgesia therapy is always interrupted [13, 15]. Therefore, the equivalent dose of fentanyl and oxycodone was 1: 60 in this study. Shin S et al. have shown that a background infusion rate of fentanyl 0.12–0.67 µg·kg^−1^·h^−1^ is safe for IV-PCA with fewer side effects [17], so we used fentanyl 8.3 μg/kg and oxycodone 0.5 mg/kg. The fentanyl-butorphanol ratio was 1: 20 [18], so we used butorphanol 0.16 mg/kg for IV-PCA. In all groups, the parameters of the IV-PCA device remain the same: total volume of 100 mL, background infusion of 2.0 mL/h, a bolus dose of 3.0 mL, lock time 15 min and infusion time of 48 h. And we provided all patients with flurbiprofen axetil 50 mg for acute pain when they returned to the ward 2 h later.

### Randomization

According to the result of the pre-experiment, 135 patients were randomized to three groups using a randomization list provided by The First Affiliated Hospital of Nanjing Medical University (computer-generated random number system), and the allocation concealment was performed using a sealed envelope by two independent researchers. To ensure blinding, an anesthesia nurse responsible for preparing IV-PCA in the PACU opened the sealed envelopes immediately after the procedure. The outcomes were recorded by another anesthetist who was blinded to the intervention. Unmasking did not occur until statistical analysis was completed.

### Outcome measures

The primary outcome measure was the recovery of bowel function, including time to flatus, time to tolerance of solids and time to defecation. We also assessed the following nine indicators, including postoperative pain intensity (total opioid consumption, number of IV-PCA boluses and 10-points visual analog scale (VAS) at 4, 10, 24, and 48 h postoperatively), sedation level with Ramsay scale, the incidence of postoperative side effects, perioperative leukocyte count, percentage of neutrophils, plasma potassium levels, time to first ambulation, patients’ satisfaction and postoperative hospital length of stay. If patients complained of pain, we recommended them to press the button of the IV-PCA device. Rescue analgesia (adjust the parameters of IV-PCA devices, and add the bolus dose) was given if the VAS score was more than 5 for 30 min. If the patients were drowsy or had respiratory depression (SpO_2_ was < 92% and respiration rate was < 10 breath/min), we would try to wake them up and supply oxygen. If the patients didn’t respond to oral commands appropriately and were persistently hypoxic, we discontinued the IV-PCA, gave reversal agents (naloxone), and excluded them from our experiment.

### Statistical Analysis

Time to flatus was considered as the primary outcome. Based on the results of the pre-experiment (15 cases in each group, 45 cases in total), the mean time to flatus was 33.0 h, 35.3 h, and 41.0 h for the three groups (Group F, Group O, and Group B), respectively.

And the standard deviations for the three groups were 8.9 h, 9.6 h, and 6.8 h, respectively. A sample size of 108 patients was found to be sufficient to detect a significant difference (α = 5%) with a statistical power (β-value) of 0.9. Considering a follow-up missed rate of approximately 20%, we needed to enroll 135 patients. The sample size was calculated with the PASS 15.0 software (Stata Corp. LP, College Station, TX, USA).

The Shapiro–Wilk test was used to determine the normal distribution of the data, and parametric statistics were applied. Homogeneity of variance was verified by the Levene test. Normally distributed data were analyzed using one-way analysis of variance (ANOVA). Continuous variables were compared using the Kruskal–Wallis H-test. Individual groups were compared using Kruskal–Wallis H-test. Repeated measurements were compared between groups using a general linear model including all time points. Simultaneously, Also, the χ^2^ test and Fisher’s exact test were used to analyze categorical variables, a *P-*value of < 0.05 was interpreted as statistically significant. SPSS 18.0 software (SPSS, Inc., Chicago, IL, USA) was used for all statistical analyses.

## Results

**Fig. 1:**
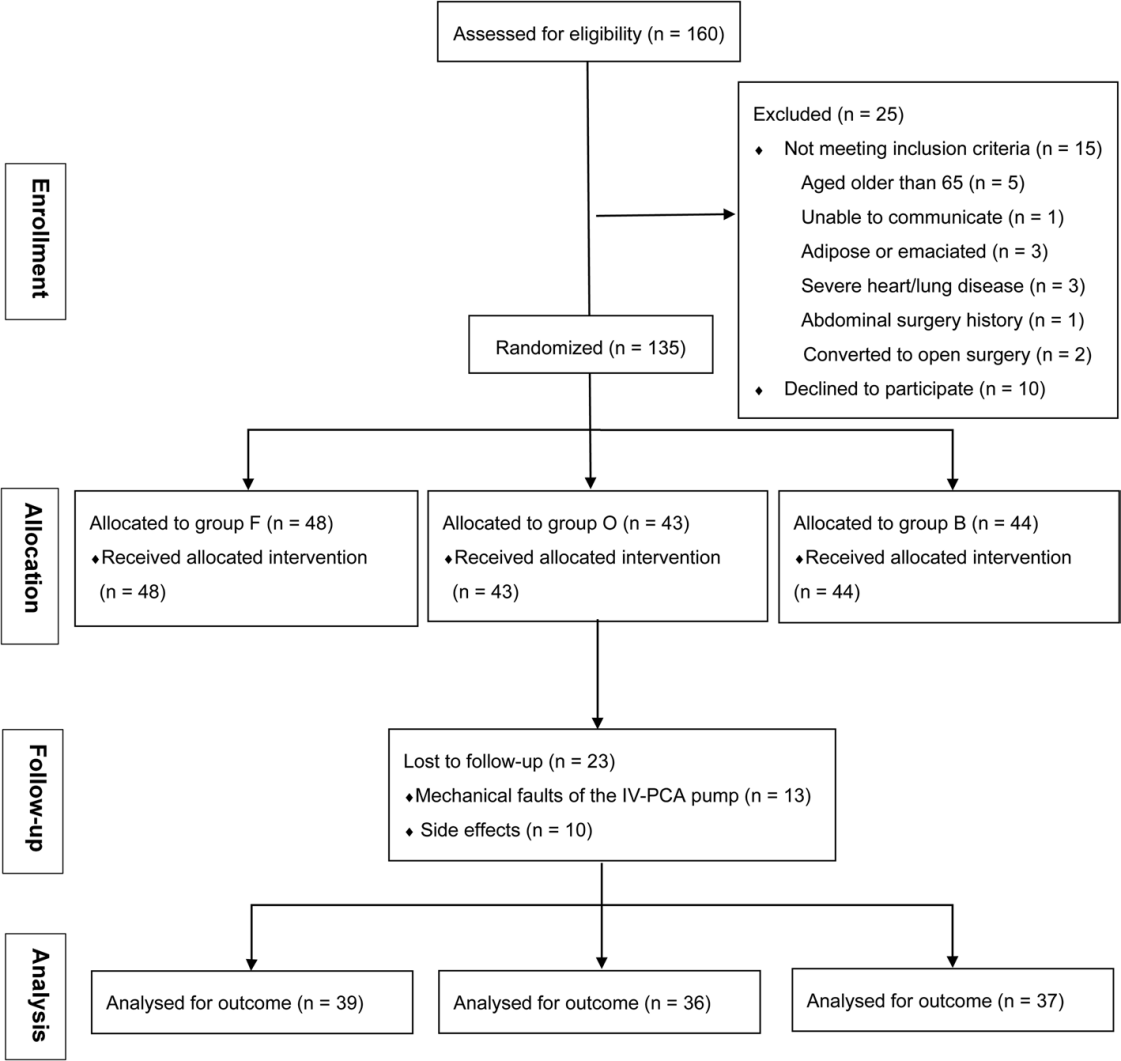
Consort flow diagram. Abbreviations: B = butorphanol; CONSORT = Consolidated Standards of Reporting Trials; F = fentanyl; IV-PCA = intravenous patient-controlled analgesia; O = oxycodone.

From March to September 2020, a total of 160 patients underwent laparoscopic hysterectomy; 15 didn’t meet the inclusion criteria and 10 declined to participate in the study. Therefore, 135 patients were first enrolled in the study, but 23 were lost to follow-up (13 were excluded due to mechanical faults of the IV-PCA pump and 10 patients were excluded for side effects). Thus, 112 patients were eventually incorporated into analysis, including 39 in group F, 36 in group O and 37 in group F (Fig. 1).

There was no significant difference in baseline patients’ characteristics, operative data, anesthesia data, consumption of intraoperative fentanyl, leukocyte count, percentage of neutrophils, and preoperative plasma potassium levels (Table 1).

**Table 1:** Patients' characteristics and other factor

	Group F (*n* = 39)	Group O (*n* = 36)	Group B (*n* = 37)
Age (y)	52.5 ± 6.6	51.4 ± 6.7	51.4 ± 6.9
Height (cm)	159.7 ± 4.6	159.1 ± 4.0	160.2 ± 4.2
Weight (kg)	60 (55–72)	60 (60–68)	60 (56–74)
BMI (kg/m^2^)	24.7 (22.1–25.8)	24.9 (23.4–26.5)	23.4 (22–25)
ASA status			
I [n (%)]	4 (10.3)	3 (8.3)	2 (5.4)
II [n (%)]	35 (89.7)	33 (91.7)	35 (94.6)
Diagnosis			
Cancer [n (%)]	18 (46.2)	15 (41.7)	18 (48.6)
CIN [n (%)]	12 (30.8)	14 (38.9)	9 (24.4)
Uterine fibroids [n (%)]	6 (15.3)	3 (8.3)	7 (18.9)
Adenomyosis [n (%)]	3 (7.7)	4 (11.1)	3 (8.1)
Duration of surgery (min)	132 (100–193)	125 (90–159)	115 (90–140)
Duration of anesthesia (min)	162 (120–235)	153(121–185)	140 (112–165)
Consumption of fentanyl (mg)	0.55 (0.50–0.65)	0.60 (0.50–0.60)	0.55 (0.50–0.60)
Total fluid (mL)	2100 (1600–2675)	2100 (1975–2500)	2000 (1600–2600)
Total loss (mL)	300 (200–507)	250 (150–400)	250 (150–388)
Leukocyte count (*10^9^/L)	5.7 (4.7–7.3)	5.3 (4.7–6.6)	5.2 (4.4–6)
Percentage of neutrophils (%)	56.3 (51.6–64.2)	56.3 (51.4–64.5)	56.5 (52.3–60.1)
Potassium (mmol/L)	3.8 (3.5–4.0)	3.8 (3.5–4.0)	3.8 (3.6–4.0)

Table legends: Normally distributed data are presented as mean ± SD, which were analyzed using ANOVA; non-normal data are presented as median (range), which were analyzed using Kruskal–Wallis H-test; categorical variables are presented as count (%), which were analyzed using the χ^2^ test and Fisher’s exact test

*Abbreviations*: *ASA *American Society of Anesthesiologists, *ANOVA * one-way analysis of variance, *BMI*  body mass index, *B*  butorphanol, *CIN*  cervical intraepithelial neoplasia, *F*  fentanyl, *O*  oxycodone, *SD*  standard deviation

The primary outcome measure was the time to flatus (measured from the end of surgery), defined as the point at which patients noticed the first bowel sound or movement [19, 20]. When noticing the first anal exhaust, patients were informed to record it or to notify nurses for judgement to ensure no data were missed. The mean time to flatus was significantly prolonged in Group B (45.2 ± 11.6 h) compared with Group F (33.1 ± 11.2 h, *P* < 0.001) and Group O (36.2 ± 10.9 h, *P* = 0.001).

**Fig. 2:**
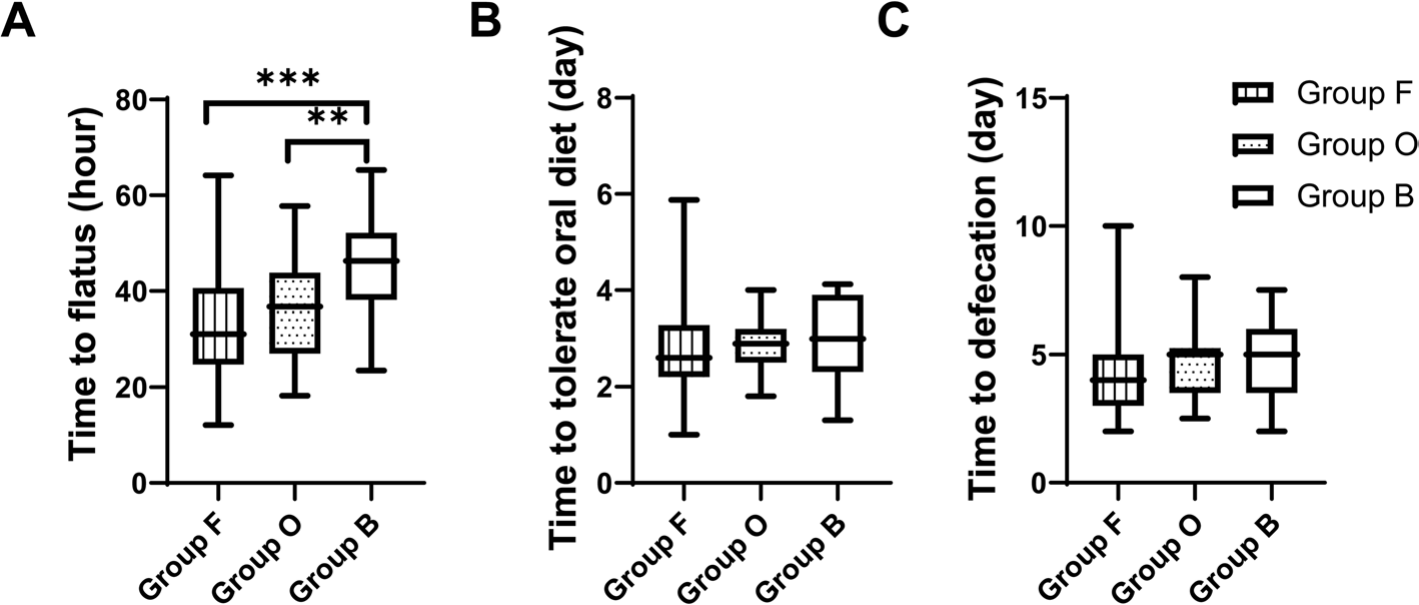
The recovery of bowel function. **A** Box plot comparing the mean time to flatus (measured from surgery). The mean time to flatus significantly longer in the group B (^***^P < 0.001, Group F versus Group B, ^**^P < 0.01, Group O versus Group B, one-way ANOVA, followed by Bonferroni’s post hoc test). **B**. Box plot comparing mean time to tolerate oral diet (measured from surgery). **C** Box plot comparing the time to defecation (measured from surgery). Abbreviations: ANOVA = one-way analysis of variance; B = butorphanol; F = fentanyl; O = oxycodone

The secondary outcomes were time to the first defecation, and time to tolerance of solids, which was defined as the patients tolerating solid food (any food requiring chewing) without vomiting or experiencing significant nausea within 4 h [19]. The mean time to tolerate oral diet was 2.9 ± 1.1 d, 2.9 ± 0.6 d, and 3.0 ± 0.8 d for Group F, Group O, and Group B, respectively. The mean time to defecation of the three groups (Group F, Group O, and Group B) prove 4.3 ± 1.7 d, 4.8 ± 1.5 d, and 4.7 ± 1.5 d, respectively. There was no significant difference in the meantime to tolerate oral diet and time to achieve defecation (Fig. 2).

Table 2 displays the analgesic outcome and the incidence of significant postoperative side effects. There was no difference in VAS, opioid consumption, and  the number of IV-PCA boluses at 4, 10, 24, and 48 h after surgery, respectively. Ramsay scores in Group B were higher than those in Group F and Group O at 4, 10, 24, and 48 h after surgery, respectively (*P* < 0.001). Patients in Group B experienced significantly more severe somnolence (*P* < 0.001) compared to Group O and Group F. The three groups witnessed a similar incidence of nausea, vomiting, and bradycardia. None of the patients included in this study experienced respiratory depression or pruritus. Overall, no difference was observed in the satisfaction score, time to first ambulation, and postoperative hospital length of stay (Table 3).

**Table 2:** Analgesic outcome and postoperative side effects

	Group F (*n* = 39)	Group O (*n* = 36)	Group B (*n* = 37)	*P-*value
Postoperative VAS pain scores				0.517
4 h	1.5 (1–3)	2 (1–4)	2 (1.5–3)	
12 h	2 (1–2.5)	2 (1–3)	2 (1.5–3)	
24 h	2 (1–2.5)	2 (1–3)	2 (1–3)	
48 h	0 (0–0.5)	0 (0–1)	0.5 (0–1)	
Total opioid consumption (mg)				0.155
4 h	7.8 (7.2–8)	7.9(7.5–9.2)	7.5 (7.1–7.8)	
12 h	30 (27.7–30)	31.3 (29.3–33)	30 (28.4–31)	
24 h	45 (43–50)	47 (43.9–50)	45 (42.5–47)	
48 h	90 (85.3–100)	92 (87.8–96)	90 (84–93)	
Number of IV-PCA boluses (times)				0.167
4 h	0 (0–0)	0 (0–1)	0 (0–0)	
12 h	0 (0–0)	0 (0–1)	0 (0–0)	
24 h	0 (0–0)	0 (0–1)	0 (0–0)	
48 h	0 (0–1)	0 (0–1)	0 (0–0)	
Postoperative Ramsay scores				< 0.001
4 h	2 (2–2)	2 (2–2)	3 (2–4)	
12 h	2 (2–3)	2 (2–2)	3 (2–4)	
24 h	2 (2–2)	2 (2–2)	2 (2–3)	
48 h	2 (2–2)	2 (2–2)	2 (2–3)	
Postoperative side effects				
Somnolence [n (%)]	1 (2.6)	0 (0)	10 (27)	< 0.001
Dizziness [n (%)]	0 (0)	2 (5.6)	5 (13.5)	0.03
Nausea [n (%)]	4 (10.3)	3 (8.3)	8 (21.6)	0.27
Vomiting [n (%)]	0 (0)	3 (8.3)	2 (5.4)	0.16
Bradycardia [n (%)]	0 (0)	0 (0)	1 (2.7)	0.65

Table legends: Non-normal data are presented as median (range), which were analyzed using Kruskal–Wallis H-test; repeated measurements were compared between groups using a general linear model including all time-points; categorical variables are presented as count (%), which were analyzed using the χ^2^ test and Fisher’s exact test

*Abbreviations*: *B*  butorphanol, *F*  fentanyl; *IV-PCA*  intravenous patient-controlled analgesia, *O*  oxycodone, *VAS*  visual analog scale

**Table 3:** The overall postoperative recovery measurement

	Group F (*n* = 39)	Group O (*n* = 36)	Group B (*n* = 37)	*P-*value
Leukocyte count (*10^9^/L)	9.3 (7.7–11.9)	9.0 (7.4–12)	9.3 (7.6–11)	0.80
Percentage of neutrophils (%)	79.5 (67.1–83.5)	79.9 (68.9–84.5)	80.9 (72.2–83.5)	0.60
Potassium (mmol/L)	3.8 (3.6–4.0)	3.8 (3.6–4.1)	3.8 (3.6–4.0)	0.85
Satisfaction	10 (10–10)	10 (9.6–10)	10 (9–10)	0.08
Time to first ambulation (h)	17.3 (15.4–19.3)	17.3 (15.9–19.6)	17.2 (15.6–19.5)	0.54
Hospital length of stay (d)	6 (5–8)	6 (5–7)	6 (5–7)	0.45

Table legends: Non-normal data are presented as median (range), which were analyzed using Kruskal–Wallis H-test

*Abbreviations*: *B*  butorphanol, *F*   fentanyl, *O*  oxycodone

## Discussion

We found that (1) fentanyl, a pure MOR agonist, had less effect on bowel function in laparoscopic hysterectomy patients; (2) oxycodone, MOR and KOR agonist showed an equivalent effect on bowel function compared to fentanyl; (3) butorphanol, a mixed opioid receptor agonist–antagonist, prolonged the recovery time of bowel function and increased the incidence of postoperative somnolence and dizziness.

Perioperative use of opioids for acute pain might cause PGID, whose clinical manifestations comprise abdominal distension, pain, nausea, vomiting, and even ileus [1]. Such symptoms considerably impair patients' recovery after surgery [1]. Currently, many comparisons of analgesic effects were made between opioids, but few studies were conducted on the effects of different opioids on bowel function [7, 13, 21, 22]. In this study, we explored the effect of the equivalent doses of pure MOR agonists, multiple opioid receptor agonists, and MOR agonist–antagonist on the recovery of bowel function in patients undergoing laparoscopic hysterectomy.

Our result suggested pure MOR agonists had less effect on bowel function. This is in disagreement with previous studies documenting that pure MOR agonists prolonged the recovery of bowel function than other opioid receptor agonists [7–9, 22]. Several reasons can explain these findings. First, we took fentanyl as the pure MOR agonist, because fentanyl enjoys fewer peripheral side effects than morphine for its lipophilic [23, 24]. Second, the equivalent dose of fentanyl and oxycodone was lower than in previous studies [22, 25]. With the oxycodone dose reduced, the incidence of postoperative nausea and vomiting remains lower. In this sense, intravenous dexamethasone and phencyclidine hydrochloride during anesthesia induction might curtail postoperative gastrointestinal side effects [26, 27]. By reducing the incidence of side effects, we improved patient compliance and reduced the rate of loss to follow-up. Third, total intravenous anesthesia was administered throughout the surgery. So, the effect of inhalation anesthesia on gastrointestinal function was ruled out [7, 28]. Forth, we found that fentanyl, oxycodone, and butorphanol allowed for equivalent analgesia. This is in agreement with previous studies. Nevertheless, we used a lower equivalent dose of fentanyl and oxycodone (1: 60) than in previous studies [22, 25]. Considering the high price of oxycodone, our analgesic regimen is reasonable to reduce the total costs related to pain management in patients. In addition, butorphanol significantly induced more severe somnolence via interaction with KOR, which is consistent with previous studies [29, 30]. In particular, the KOR agonist-related side effects were significantly greater in women than men [31, 32]. Thus, patients treated with butorphanol were reluctant to engage in postoperative physical activities, which would prolong the recovery of bowel function [33].

We showed that butorphanol, the MOR opioid receptor agonist–antagonist, has no advantage in the occurrence of PGID compared with fentanyl, the pure MOR agonists. We, therefore, speculated that PGID is not only caused by MOR but also by other opioid receptors. Thus, further work on the pharmacological mechanism of opioid receptors in enteric neurons systems is needed. And to avoid PGID, we need multimodal anesthesia and analgesia based on opioid-free or opioid-sparing regimens to reduce perioperative opioid consumption [34, 35].

Some limitations also stand out in the present study: (1) butorphanol might have higher efficacy for KOR in females than in males [31, 32]. This study had limited samples and was female-only; therefore, further studies are needed in a large cohort of male and female individuals; (2) we gave a large dose of fentanyl intraoperatively in all three groups, and the elimination/clearance half-life of fentanyl was 2–4 h [36], so the effect of high-dose fentanyl on postoperative bowel function may be disruptive. Thus, further studies may use multimodal analgesia protocols to limit the consumption of perioperative opioid drugs; (3) postoperative gastrointestinal function evaluation criteria are subjective. Although “time to flatus” was adopted by many clinical trials [19, 37], there is still a need for systematic and objective criteria for evaluating gastrointestinal function.

## Conclusion

This study showed butorphanol prolonged the recovery of bowel function with more severe somnolence and dizziness, while fentanyl, a pure MOR agonist, interfered much less with bowel function. This study indicated butorphanol, a mixed opioid receptor agonist–antagonist, is not well suitable for IV-PCA in patients undergoing laparoscopic hysterectomy.

## Data Availability

The data are not available for public access because of patient privacy concerns, but are available from the corresponding author on reasonable request.

## Abbreviations

ASA: American Society of Anesthesiologists
ANOVA: One-way analysis of variance
BIS: Bispectral Index
CONSORT: Consolidated Standards of Reporting Trials
DOR: Delta-opioid receptors
EKG: Electrocardiography
HR: Heart rate
IV-PCA: Intravenous patient-controlled analgesia
KOR: Kappa-opioid receptors
MOR: Mu-opioid receptors
MBP: Mean blood pressure
PGID: Postoperative gastrointestinal tract dysfunction
PACU: Post-anesthesia care unit
SpO_2_: Oxygen saturation
VAS: Visual analogue scale
